# Comparison of per operative methotrexate infusion with postoperative intra silicon oil methotrexate injections for prevention of proliferative vitreoretinopathy development after vitrectomy for rhegmatogenous retinal detachment repair

**DOI:** 10.12669/pjms.42.3.13344

**Published:** 2026-03

**Authors:** Ahmad Zeeshan Jamil, Muhammad Hannan Jamil, Abdullah Muaz, Hina Nazir

**Affiliations:** 1 Ahmad Zeeshan Jamil, MBBS, MCPS, FCPS, FRCS, FCPS (VRO), MHPE Professor of Ophthalmology, Sahiwal Teaching Hospital and Sahiwal Medical College, Sahiwal, Pakistan; 2 Muhammad Hannan Jamil, MBBS, MCPS, FCPS, FRCS, FCPS (VRO) Assistant Professor of Ophthalmology, Ali Fatima Teaching Hospital and Abu Amara Medical College, Lahore, Pakistan; 3 Abdullah Muaz, MBBS, FCPS, FRCS Senior Registrar Ophthalmology, Sahiwal Teaching Hospital and Sahiwal Medical College, Sahiwal, Pakistan; 4 Hina Nazir, MBBS, FCPS, FRCS Senior Registrar Ophthalmology, Sahiwal Teaching Hospital and Sahiwal Medical College, Sahiwal, Pakistan

**Keywords:** Methotrexate, Proliferative Vitreoretinopathy, Retinal detachment, Silicon oil, Vitrectomy

## Abstract

**Objective:**

To compare the efficacy of repeated intrasilicon oil methotrexate injections with methotrexate infusion during pars plana vitrectomy in preventing proliferative vitreoretinopathy (PVR) development.

**Methodology:**

This randomized controlled trial was conducted at Sahiwal Teaching Hospital, Sahiwal and Ali Fatima Teaching Hospital Lahore from March 2024 to May 2025. Study included patients of rhegmatogenous retinal detachment of both genders from 20 to 70 years of age. Patients were divided into two groups with 60 subjects in each group. Group one was regarded as control group where per operative methotrexate (MTX) infusion was used. Group two was regarded as the study group that received repeated injections of intra-silicon oil MTX. Primary outcomes were proliferative vitreoretinopathy (PVR) development and retinal re-detachment at three months. Secondary outcome was visual acuity improvement.

**Results:**

There were 66(55%) male and 54(45%) female patients. Postoperatively visual acuity improved in 48(80%) cases in group one and 44(73.3%) cases in group two. PVR developed in 6(10%) patients in group one while no patient developed PVR in group two. Recurrent retinal detachment occurred in 8(13.3%) cases in group one and 2(3.3%) cases in group two. Chi-square test showed that there was statistically significant difference between the two groups in the development of PVR and retinal re-detachment (p<0.05).

**Conclusion:**

Repeated intra-silicon oil injections of MTX are more effective than intraoperative infusion of MTX in preventing the development of PVR and decreasing the rate of retinal re-detachment.

**Registration No.: NCT:** 06289205.

## INTRODUCTION

Retinal detachment (RD) is one of the major causes of irreversible blindness.^1^ Rhegmatogenous retinal detachment (RRD) is the most common form of retinal detachment where a full thickness break in neurosensory retina allows passage of fluid and separation of sensory retina from the pigment epithelium. Incidence of RRD has increased in the recent years globally. Age related changes in the vitreous like posterior vitreous detachment, the increasing prevalence of high myopia and growing number of cataract surgeries performed worldwide, have been implicated as major risk factors.^2^-^4^

With increase in expertise of surgeons and refined gadgets, pars plana vitrectomy (PPV) is the most performed surgical procedure for the treatment of RRD.^5^ Proliferative vitreoretinopathy (PVR) poses a significant barrier in achieving satisfactory anatomical and physiological outcome after retinal detachment surgery. PVR formation occurs because of healing reaction initiated by retinal detachment. There is fibro-cellular response in which pigment epithelial cells, glial cells and fibroblasts take part in forming contractile membranes on both surfaces of the sensory retina, resulting in retinal shortening and stiffness. PVR is identified as the most common cause of failed retinal detachment surgery and recurrent retinal detachment, reported in up to 5-10% of cases despite initial successful repair.^6^ While the incidence of PVR in subsequent failed RD surgeries is as high as 75%.^7^

Various preventative strategies, both surgical and pharmacological, have been explored to address the challenge of PVR. New surgical techniques are aimed at minimizing dispersion of retinal pigment epithelial cells. Various pharmacological agents are used to inhibit cell proliferation, migration, or inflammation. Corticosteroids, anti-proliferative drugs, anti-neoplastic medications, vascular endothelial growth factor inhibitors, non-steroidal anti-inflammatory drugs and low molecular weight heparin have been studied in that regard. But there is no universally accepted method for the complete prevention of PVR.^8^ It is utmost important to find effective ways to prevent formation of PVR to improve outcome after RD repair.

Methotrexate (MTX), a folate antagonist, is such an agent that has gained attention in ophthalmology. It has dual anti-inflammatory and anti-proliferative properties. It competitively inhibits key enzymes involved in DNA synthesis, therefore suppressing cellular proliferation. Moreover, MTX exhibits anti-fibrotic effects, thereby interfering with various stages of PVR development. It also inhibits abnormal cell growth and glial tissue formation. MTX has been utilized by different routes and in different regimen during and after RD surgery.^9^,^10^ It has shown encouraging results for prevention of PVR development. Intraoperative MTX infusion and repeated post operative MTX injections have proven effective.^10^ While various studies have shown safety and effectivity of MTX there is a shortage of studies directly comparing different treatment approaches.^11^ The current study compares peroperative methotrexate infusion with postoperative intra-silicon oil methotrexate injections for prevention of PVR development after vitrectomy for RRD repair.

## METHODOLOGY

This single blinded randomized controlled study was conducted at Sahiwal Teaching Hospital, Sahiwal and Ali Fatima Teaching Hospital Lahore from March 2024 to May 2025.

### Ethical Approval:

The study followed the ethical standards set by the Declaration of Helsinki. Registration number of study was NCT**:** 06289205. The study protocol was approved by institutional review boards of participating hospitals under approval letters 60/IRB/SLMC/SWL dated April 28, 2023 and RERC-10-2023-AFH-01 dated October 16, 2023. Written informed consent was taken from all the subjects.

### Inclusion & Exclusion Criteria:

In this study two different Methotrexate (MTX) delivery protocols in preventing proliferative vitreoretinopathy (PVR) development after pars plana vitrectomy (PPV) for rhegmatogenous retinal detachment (RRD) repair were compared. The study included patients of both sexes between 20 and 70 years of age. Exclusion criteria included the presence of PVR grade C, history of previous retinal detachment surgery, penetrating ocular trauma, intraocular foreign bodies, previous glaucoma filtration surgery, allergy to methotrexate, and pregnant and lactating women. A detailed history of the patients’ presenting complaints, duration of symptoms, and risk factors for retinal detachment were obtained. Comprehensive ocular examination, including visual acuity, intraocular pressure, and examination of the anterior and posterior segments, was performed. The extent of retinal detachment, location of retinal breaks, and presence of PVR were documented.

The sampling method was simple random sampling and sample size was calculated by using online software. Using lottery methods patients were divided into two groups with 60 subjects in each group. Group one was regarded as control group where per operative methotrexate infusion was used by mixing 75 mg of methotrexate into one liter of balanced salt solution (BSS). Group two was regarded as the study group that received 500 µg of intra-silicon oil methotrexate at the end of the surgery and then at 1^st^, 2^nd^, 3^rd^, 4^th^ and 6^th^ post operative weeks.

Standard 23 Guage pars plana vitrectomy was performed by two surgeons, one surgeon at Sahiwal Teaching Hospital Sahiwal and other surgeon at Ali Fatima Teaching Hospital Lahore. Silicon oil 1000 cSt was used as post operative tamponade in all subjects. A combination of steroid and antibiotic eye drops was administered eight times per day during the first postoperative week. The dosing was tapered off during the next three weeks. Patients were instructed to adopt the appropriate posture according to the location of retinal breaks.

Follow-up was performed every week for three months. The development of PVR, occurrence of retinal detachment, intraocular pressure, and visual acuity were noted. The primary outcomes were development of PVR or the recurrence of retinal detachment. All patients were followed up until the development of one of the primary outcomes or completion of three-month follow-up, whichever occurred earlier. Improvement in visual acuity was secondary outcome of the study. Final visual acuity was measured at completion of three-month follow up or meeting the primary outcome, whichever was earlier.

All information was collected using a specially designed proforma and entered into SPSS version 26. Qualitative variables, such as sex, PVR development, recurrence of retinal detachment, and improvement in visual acuity, were presented as frequencies and percentages. Quantitative variables, such as age and intraocular pressure, were presented as mean and standard deviation. Pearson’s chi-square test was used for comparative analysis between two groups. Statistical significance was set at P≤0.05.

## RESULTS

One hundred and twenty patients were included in the study with an equal distribution between the two groups. Study included 66(55%) male and 54(45%) female patients. Right eye was involved in 56(46.7%) and left eye was involved in 64(53,3%) patients. Retinal detachment involving four, three, two and one quadrants was found in 53.3%, 16.7%, 16.7% and 13.3% respectively. At presentation macula was detached in 84.2% patients.

Baseline and final visual acuity at three months are shown in Table-I. Visual acuity improved in 48(80%) cases in group one and 44(73.3%) cases in group two postoperatively. There was no statistical difference between two groups in baseline and final visual acuity with p value 0.37 and 0.45 respectively. In methotrexate infusion group there was statistically significant difference in baseline and final visual acuity with p=0.000. Likewise, there was statistically significant difference in baseline and final visual acuity with p=0.000 in methotrexate injection group.

**Table-I:** Baseline and Final Visual Acuity in Two Groups.

Visual acuity	Baseline visual acuity		Final visual acuity	
	MTX infusion	MTX injection	MTX infusion	MTX injection
6/18 to 6/6	1(1.7%)	1(1.7%)	3(5%)	5(8.3%)
6/24 to 6/60	6(10%)	11(18.3%)	30(50%)	35(58.3%)
FC to HM	27(45%)	30(50%)	24(40%)	16(26.7%)
Less than HM	26(43.3%)	18(30%)	3(5%)	4(6.7%)

After PPV development of PVR was noted in 6(10%) patients in group one while no PVR development was documented in group two. Statistically significant difference was noted when comparing the development of PVR between the two groups with p=0.01.

At the end of three months re-detachment of retina was noted in 8(13.3%) patients in group one and 2(3.3%) patients in group two. Difference between the groups was statistically significant with p=0.048.

## DISCUSSION

This randomized controlled trial provides the first direct comparison between methotrexate (MTX) infusion and repeated postoperative MTX injections for the prevention of proliferative vitreoretinopathy (PVR) after pars plan vitrectomy (PPV) for rhegmatogenous retinal detachment (RRD). Outcomes were assessed in terms of PVR development, retinal re-detachment and postoperative visual improvement.

Our results showed that frequency of PVR development was significantly lower in MTX injection group than MTX infusion group. At the end of three-month follow-up, there was no case of PVR development in MTX injection group while 6(10%) patients developed PVR in MTX infusion group. As PVR has major role in retinal detachment surgery failure, the absence of PVR development in injection group is clinically significant.^12^,^13^ PVR develops during 60 to 90 days postoperative period after retinal detachment surgery.^14^ Infusion of MTX during retinal detachment surgery provides a loading dose.^15^ Retinal tissue absorbs MTX that is sufficient to stop development of PVR for the immediate postoperative period. But long-term prevention is not provided. Intravitreal half-life of MTX is three to five days.^16^ Therefore, repeated doses of MTX during the first two to three months postoperatively are needed to give long term prevention against the development of PVR.^17^ It underscores the superiority of MTX injections in prevention of PVR development. Probably it is due to prolonged availability of MTX because of repeated injections during the critical period of cellular proliferation and membrane formation. Current study addressed the lack of head-to-head comparison between single intraoperatavie MTX infusion and repeated postoperative injections of MTX. Our research suggests a shift in prophylaxis strategy from a single intraoperative dose to a planned postoperative dosing schedule. This protocol better matches the drug delivery to the period of highest PVR risk.

Anatomical outcome, as measured by the frequency of retinal re-detachment, was different between the two groups. Re-detachment occurred in 2(3.3%) patients in MTX injection group as compared to 8(13.3%) patients in MTX infusion group. Lower frequency of re-detachment in MTX injection group is likely due to inhibition of fibro cellular membrane formation and contraction thereof. Our findings are in alignment with the findings of a study conducted by Hughes and coauthors. They gave repeated intravitreal methotrexate injections postoperatively and achieved 90% rate of anatomical success.^18^ These findings suggest that adjunctive use of methotrexate in retinal detachment surgery effectively prevents the development of PVR and retinal re-detachment.^19^,^20^

Numerically there were more patients in MTX injection group who achieved visual outcomes in the range of 6/24-6/6. But there was no statistically significant difference between the two groups. It shows that whether MTX is infused intraoperatively or injected postoperatively, it has no adverse effect on visual recovery.^21^ Our findings are consistent with the findings of the study conducted by Jamil et al.^22^ Absence of significant difference in post operative visual recovery between the two groups can be due to many factors including macular status, duration of retinal detachment, extent of retinal detachment and ocular comorbidities. In our study majority of patients in both groups presented with detachments involving macula. This factor might be limiting good visual recovery regardless of choice of treatment strategy. Moreover, short follow up did not allow to record the full vision recovery. Following RRD repair, visual recovery continues beyond three months.^23^

### Strengths of this study:

The strengths of this study include its randomized controlled design, adequate sample size, and dual-center execution. All of these enhance the generalizability and reliability of the research.

### Limitations:

A primary limitation is the short-term follow-up. Studies with longer follow up are required to validate these findings. Longer follow up can better assess the impact of methotrexate use in PVR prevention, improved visual function and rate of successful retinal surgery. Future research should be conducted to determine optimal dosing schedule. Moreover, combined use of MTX with other adjunctive therapies should be explored in cases of high risk RRD.

Overall, current study supports postoperative MTX injections as a more effective prophylactic approach than intraoperative infusion. This evidence provides a clinically relevant strategy to reduce PVR and improve surgical success in retinal detachment repair.

## CONCLUSION

Repeated intra-silicon oil injections of MTX are more effective than intraoperative infusion of MTX in preventing the development of PVR and decreasing the rate of retinal re-detachment. This approach is not associated with adverse visual outcome and results in improved postoperative visual acuity.

### What is known:

We know that methotrexate can help prevent development of proliferative vitreoretinopathy (PVR) after pars plana vitrectomy for rhegmatogenous retinal detachment surgery.

### What is unknown:

Which delivery method of methotrexate, intraoperative infusion versus repeated postoperative injections, works better. Comparative effectiveness of different methotrexate delivery protocols especially Intra-operative infusion versus postoperative repeated injections remains undetermined.

### What this study adds:

This is the first randomized controlled trial that compares methotrexate delivery protocols, per operative infusion with repeated postoperative injections, to address the gap of comparative evidence.

Findings can potentially shift prophylactic strategy from single intraoperative use to a structured postoperative regimen. That will align drug delivery with high-risk period for PVR development.

### Author`s Contribution:

**AZJ:** Concept and design of the study, Manuscript drafting, Responsible and accountable for the accuracy or integrity of the work.

**MHJ:** Acquisition of data, Critical revision,

**AM:** Design of study, Manuscript drafting,

**HN:** Acquisition and analysis of data, Drafting of manuscript

All authors have read and approved the final version of the manuscript.
